# Effects of Cellulase and β-Glucosidase on the Quality and Aroma Profiles of Crown Pear Wine

**DOI:** 10.3390/foods15213843

**Published:** 2026-10-27

**Authors:** Xiaoxuan Bai, Zhenzhen Liu, Fengjiao Xin, Yang Song, Xiaobin Feng, Yanli Jia, Qiqi Chen, Kangzhuang Zhang, Fang Zhang, Ze Wang, Xinyu Xie, Huizhuo Ji

**Affiliations:** 1 Cangzhou Academy of Agriculture and Forestry Sciences, Cangzhou 061001, China; baixiaoxuan9754@163.com (X.B.);; 2 Institute of Food Science Technology Nutrition and Health (Cangzhou), Chinese Academy of Agricultural Sciences, Cangzhou 061000, China; 3 Laboratory of Biomanufacturing and Food Engineering, Institute of Food Science and Technology, Chinese Academy of Agricultural Sciences, Beijing 100193, China; 4 Cangzhou Municipal Bureau of Agriculture and Rural Affairs, Cangzhou 061001, China

**Keywords:** crown pear wine, cellulase, β-glucosidase, aroma compounds, sensory evaluation, comprehensive evaluation

## Abstract

Enzymatic treatment is an effective approach for regulating the quality and aroma of fruit wines. In this study, the effects of cellulase and β-glucosidase at different concentrations on pear wine quality, volatile aroma compounds, and sensory characteristics were investigated. Multivariate statistical analyses and entropy-weighted TOPSIS were used to characterize aroma differentiation and rank treatments based on comprehensive quality attributes. Enzymatic treatment significantly affected clarification, color, antioxidant capacity, and volatile aroma profiles, whereas most conventional physicochemical parameters showed limited changes. A total of 54 and 53 volatile compounds were identified in cellulase- and β-glucosidase-treated wines, respectively. Cellulase treatment produced a greater increase in esters and enhanced fruity aroma, whereas β-glucosidase markedly promoted terpenes and their derivatives, which were associated with enhanced floral aroma. Notably, linalool increased by 261.76–617.23% in cellulase-treated wines, suggesting its potential contribution to floral aroma enhancement. PCA and OPLS-DA demonstrated distinct volatile profiles between the two enzyme-treatment systems, with enzyme type exerting a greater influence than enzyme concentration. Entropy-weighted TOPSIS identified 1.25 g/L cellulase and 10 g/L β-glucosidase as the highest-ranked treatments among the tested concentrations for the two enzymes, respectively. These findings highlight the distinct roles of the two enzymes in modulating pear wine quality and aroma.

## 1. Introduction

Pears are fresh, tender, and juicy, with a pleasant sweet-and-sour taste and rich nutritional value. They are widely cultivated around the world, are well-suited for processing, and serve as an important raw material for winemaking [1]. In recent years, fruit wines have attracted increasing attention as alternatives to traditional grape wine because they provide opportunities for the utilization of diverse fruit resources and the development of products with distinctive sensory characteristics [2]. However, compared to common winemaking fruits such as grapes and apples, pears contain fewer aroma compounds and have a milder flavor. As a result, pear wine often exhibits poor flavor and a thin mouthfeel, resulting in limited market competitiveness [3]. Improving the flavor quality of pear wine is therefore of great significance to the development of the pear wine industry.

The aroma characteristics of fruit wine are determined by the composition and interactions of volatile compounds generated or released during processing and fermentation. In fruit matrices, aroma-related compounds may occur in both free and bound forms, and the release of aroma precursors can therefore influence the final volatile profile of fermented beverages [4]. Current strategies for enhancing fruit wine aroma include the use of selected microorganisms capable of producing aroma compounds and the application of exogenous enzymes to facilitate the release or transformation of aroma-related substances [5,6]. Compared with strategies relying exclusively on microbial metabolism, enzymatic treatment provides a direct approach for modifying the fruit matrix before or during fermentation. In particular, enzymes capable of degrading structural or glycosidic components of plant tissues may facilitate the release of intracellular compounds or bound aroma precursors, thereby potentially altering the composition and perception of fruit wine aroma [7].

Cellulase promotes the release of intracellular substances by hydrolyzing cellulose in the cell wall, thereby influencing the aroma compounds in fruit juice [8]. Jiang et al. [8] found that adding cellulase to hawthorn juice reduces undesirable flavor compounds, such as tert-butyl methyl ether, and increases aroma compounds, such as ethyl formate, thereby optimizing the odor profile of hawthorn juice. Wang et al. [9] found that adding pectinase and cellulase to sea buckthorn pomace can increase the levels of esters and alcohols in the pomace. Nevertheless, most available studies have focused on juice extraction, fruit pomace utilization, or individual aroma-related changes, while the effects of cellulase concentration on the integrated quality of fruit wine remain insufficiently characterized.

β-Glucosidase provides a different enzymatic approach to aroma regulation. It can hydrolyze bound glycosides in fruit juice, releasing free glycoside moieties that promote the formation of aroma compounds, thereby enhancing the wine’s aroma [10]. A study in which grape and cherry juices were treated with various commercially available enzyme preparations found that the preparation containing β-glucosidase as its main component was most effective at enhancing the aroma of the juices. By increasing the levels of terpenes, higher alcohols, and esters, it significantly enhanced the floral and fruity aromas of the juices [11]. Bai et al. [12] found that adding both free and immobilized β-glucosidase before fermentation significantly increased the levels of alcohols, esters, terpenes, and their derivatives in Xiahei wine. These studies demonstrate the potential of β-glucosidase for enhancing the release of aroma-related compounds in fruit wines.

However, the response to β-glucosidase treatment may differ from that induced by cellulase because the two enzymes act on different components of the fruit matrix. Meanwhile, changes induced by enzymatic treatment are not necessarily restricted to aroma. The addition of enzymes may also influence clarification, color, phenolic and flavonoid contents, and antioxidant properties, suggesting that enzyme treatment should be evaluated from a multidimensional quality perspective rather than solely according to volatile compound production [9,13]. However, direct comparisons of cellulase and β-glucosidase across different concentrations within the same fruit fermentation system remain limited, particularly regarding their differential effects on aroma compounds and multiple quality attributes. Furthermore, there has been little research to date on using enzymes to improve the quality of pear wine. A direct comparison of their effects under different treatment concentrations is therefore necessary to clarify their respective contributions to pear wine quality and aroma.

Based on the different catalytic functions of the two enzymes, we hypothesize that cellulase and β-glucosidase will have different effects on the volatile aroma characteristics and overall quality of the fermented Crown Pear beverage, and that these effects may be related to enzyme concentration. Accordingly, this study investigated the effects of cellulase and β-glucosidase at different concentrations on the multiple quality and aroma characteristics of crown pear wine and to clarify the differential effects of the two enzymes. The findings are expected to provide a more comprehensive understanding of the differential effects of cellulase and β-glucosidase on pear wine and to provide a basis for targeted enzyme selection and concentration optimization in pear wine production.

## 2. Materials and Methods

### 2.1. Experimental Materials and Reagents

‘Crown’ pears at commercial maturity were purchased in February 2026 from Yiming Xianshiji Supermarket in Cangzhou, Hebei Province, China. Fruits with uniform appearance and without visible defects were selected for the experiment.

Potassium metabisulfite (food grade; Zibo Changcheng Chemical Factory, Zibo, China), methylene blue (Tianjin Dengfeng Chemical Reagent Factory, Tianjin, China), sodium potassium tartrate tetrahydrate (Tianjin Dengfeng Chemical Reagent Factory, Tianjin, China), phenolphthalein (Yuan Ye Biotechnology Co., Ltd., Shanghai, China), sodium nitrite (McLean Biochemical Technology Co., Ltd., Shanghai, China), aluminum chloride (McLean Biochemical Technology Co., Ltd., Shanghai, China), copper sulfate pentahydrate (McLean Biochemical Technology Co., Ltd., Shanghai, China), glucose (McLean Biochemical Technology Co., Ltd., Shanghai, China), gallic acid (McLean Biochemical Technology Co., Ltd., Shanghai, China), rutin (McLean Biochemical Technology Co., Ltd., Shanghai, China), and 4-methyl-2-pentanol (purity 99%; McLean Biochemical Technology Co., Ltd., Shanghai, China) were used in this study.

Commercial cellulase (derived from *Trichoderma reesei*; declared activity, 3500 U/g; Cangzhou Xiasheng Enzyme Biotechnology Co., Ltd., Cangzhou, China) and β-glucosidase (derived from *Aspergillus niger*; declared activity, 1000 U/mL; Cangzhou Xiasheng Enzyme Biotechnology Co., Ltd., Cangzhou, China) were used. The *Saccharomyces cerevisiae* (Vivance, Angela Yeast Co., Ltd., Yichang, China) was used for alcoholic fermentation.

### 2.2. Experimental Methods

#### 2.2.1. Crown Pear Wine Brewing Process

Crown pears were sorted, washed, cut into pieces, and juiced. For each treatment, 28 kg of pears were processed, yielding approximately 16 L of pear juice. Before adjustment, the initial must had a pH 4.82, soluble solids content of 12.7 °Brix, and titratable acidity of 1.44 g/L (expressed as tartaric acid). Sucrose was then added to adjust the soluble solids content to 18 °Brix. Potassium metabisulfite was added at a calculated amount equivalent to 60 mg/L SO_2_ to prevent oxidation; the 60 mg/L value refers to the theoretical SO_2_-equivalent addition. The prepared pear juice was pasteurized at 60 °C for 10 min, then left to cool to room temperature.

Enzyme treatment: Following the method described by Ozyilmaz [13] and in accordance with the product’s recommended usage instructions, cellulase and β-glucosidase were separately added to pear juice at concentrations of 1.25 g/L, 2.5 g/L, 5 g/L, 7.5 g/L, and 10 g/L to pear juice. The cellulase-treated groups (C) were designated C1–C5, and the β-glucosidase-treated groups (G) were designated G1–G5, corresponding to the five enzyme concentrations, respectively. A control group (CK) without added enzymes was established, and the samples were enzymatically hydrolyzed for 2 h at 50 °C.

**Figure 1:**
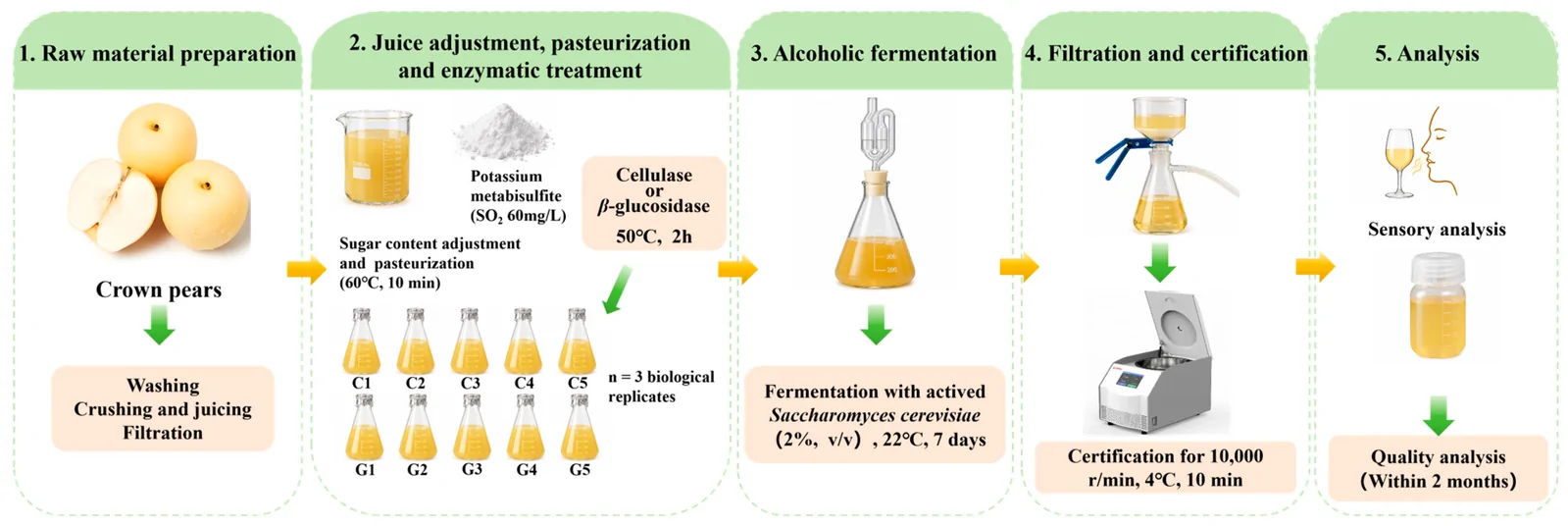
Flowchart.

Following enzyme treatment, 400 mL of pear must from each treatment was transferred into a 500 mL glass Erlenmeyer flask fitted with a silicone stopper and a one-way valve. Activated *Saccharomyces cerevisiae* was inoculated at 2% (*v*/*v*), and alcoholic fermentation was conducted at 22 °C for 7 days. Fermentation progress was monitored using the CO_2_ weight-loss method. Fermentation was considered complete when the weight loss between consecutive measurements remained below 0.2 g/100 mL for two consecutive days, accompanied by cessation of bubble formation and stabilization of the alcohol content. Each treatment was independently fermented in triplicate. Subsequently, the samples were centrifuged at 10,000 r/min and 4 °C for 10 min and filtered to remove sediments, and the crown pear wines were obtained. Samples used for sensory evaluation were transferred into glass bottles and stored at 4 °C for 2 days before evaluation. The remaining samples for physicochemical, antioxidant, color, and volatile compound analyses were aliquoted and stored at −20 °C until analysis. Different quality parameters were analyzed in batches, and all analyses were completed within two months after fermentation. The production process and subsequent sample preparation procedures are illustrated in Figure 1.

#### 2.2.2. Determination of Physical and Chemical Parameters

Ethanol volume fraction, reducing sugars, and total acidity were determined according to the Chinese National Standard: GB 5009.225—2023 [14]. The sample was distilled, and the alcohol content of the distillate was determined using the pycnometer method. Soluble solids (°Brix) were determined by refractometry using a handheld refractometer.

Total phenols were determined using the Folin-phenol method [15].

Total flavonoids were determined using the sodium nitrite–aluminum chloride colorimetric method, based on the method described by Wang et al. [15].

#### 2.2.3. Determination of Antioxidant Capacity

The iron ion reduction capacity (FRAP) was determined using the method described by Zou et al. [16]: Take 0.1 mL of the sample and 3.9 mL of the TPTZ working solution, mix thoroughly, and allow to react for 10 min. Measure the absorbance at 593 nm. Based on the standard curve generated using FeSO_4_ at various concentrations, the results are expressed as FRAP values.

The DPPH radical scavenging capacity was determined using the method described by Wang et al. [15], with minor modifications: Mix 1 mL of the sample with 3 mL of DPPH ethanol solution and let it stand for 30 min at room temperature. Measure the absorbance of the sample at 517 nm. Calculate the result using the following formula:

$$\mathrm{DPPH}\;\mathrm{RSA}\;(\%)=(1-\frac{A_{\mathrm{sample}}-A_{\mathrm{control}}}{A_{\mathrm{blank}}})\times 100$$

The ABTS radical scavenging capacity was determined using the method described by Wang et al. [15], with minor modifications: with minor modifications: Mix 7 mmol/L ABTS with 2.45 mmol/L potassium persulfate in a 1:1 (volume ratio) mixture and incubate in the dark for 16 h. Dilute the solution with phosphate buffer until the absorbance at 734 nm is 0.70 ± 0.02; mix with the sample, incubate in the dark for 6 min, and then measure the absorbance at 734 nm. Calculate the result using the following formula:

$$\mathrm{ABTS RSA}\;(\%)=(1-\frac{A_{\mathrm{sample}}-A_{\mathrm{control}}}{A_{\mathrm{blank}}})\times 100$$

#### 2.2.4. Determination of Color and Clarity Parameters

The color characteristics of pear wine samples were determined using spectrophotometric methods according to the International Organisation of Vine and Wine (OIV). The CIE L*, a*, and b* values were determined according to the OIV-MA-AS2-11 method [17]. Briefly, the transmittance spectra of the samples were recorded from 380 to 780 nm at 5 nm intervals using distilled water as the reference. The measurements were performed with the D65 standard illuminant and a 10° standard observer, and the CIE L*, a*, and b* coordinates were calculated from the spectral transmittance data.

Color intensity and shade were determined separately according to the conventional OIV-MA-AS2-07B method [17]. Absorbance values were measured at 420, 520, and 620 nm using a 1-cm optical path. Color intensity was calculated as the sum of the absorbance values at 420, 520, and 620 nm, whereas shade was calculated as the ratio of absorbance at 420 nm to that at 520 nm.

Clarity was assessed by measuring the transmittance (%) of the fermented pear beverage at 680 nm using the method described by Yang et al., with distilled water as the reference, by measuring the transmittance of the pear wine at 680 nm [18].

#### 2.2.5. GC-MS Analysis of Aroma Compounds

Aroma compounds in pear wine were determined using headspace solid-phase microextraction (HS-SPME) (InLab PAL RSI, Zhida Scientific, Guangzhou, China) (SPME fiber: DVB/CAR/PDMS) coupled with a GCMS-QP2020 NX gas chromatography–mass spectrometry (Shimadzu Corporation, Kyoto, Japan) [12]. A total of 5 mL of pear wine was placed in a headspace vial, followed by the addition of 1.5 g of sodium chloride and 20 μL of internal standard solution (4-methyl-2-pentanol, 0.3244 μg/mL). The samples were equilibrated and extracted at 55 °C for 40 min with agitation at 1800 r/min before GC-MS analysis.

GC-MS analysis was performed using an SH-I-5Sil MS capillary column (30 m × 0.25 mm × 0.25 μm, Shimadzu Corporation, Kyoto, Japan). The SPME fiber was thermally desorbed in the injection port at 270 °C for 3 min under splitless mode. The oven temperature was initially set at 40 °C and maintained for 2 min, then increased to 230 °C at a rate of 4 °C/min and held for 5 min. Helium was used as the carrier gas at a constant flow rate of 1.3 mL/min. The mass spectrometer was operated in electron ionization (EI) mode at 70 eV, with the ion source temperature maintained at 250 °C, and the mass scan range was set from *m*/*z* 29 to 500. Aroma compounds were identified by comparing their mass spectra with the NIST 20 library (match threshold > 80%). Retention-index confirmation was not performed; therefore, compound assignments should be regarded as putative identifications based on mass-spectral similarity. The concentration of each compound was calculated by comparing the peak area of the compound with that of the internal standard. The resulting values represent semi-quantitative estimates rather than absolute concentrations.

In view of the fact that the analytes were semi-qualitative, here the estimated odor activity value (EOAV) was used, which was calculated by dividing the concentration of the compound by the odor threshold value taken from the literature [19,20,21,22,23,24,25,26,27,28].

#### 2.2.6. Sensory Analysis

Sensory evaluation was conducted by a trained panel consisting of 10 members (5 males and 5 females, aged 25–45 years) with previous experience in fruit wine sensory evaluation. Before formal evaluation, the panelists participated in several training sessions to familiarize themselves with the sensory attributes and evaluation procedures. During training, reference standards representing fruity, floral, and overall aroma characteristics were discussed to establish a consistent sensory vocabulary among panelists. The selected attributes were based on the characteristic aroma profiles of pear wine and previous studies on fruit wine sensory evaluation.

In each round of evaluation, samples are randomly coded and presented in a random order to minimize evaluation bias. Each sample, approximately 20 milliliters in volume, was placed in an odorless glass cup and served at a temperature of 20 °C. Tasters were instructed not to swallow the samples and rinse their mouths with water between samples. Each sample was evaluated independently using a 10-point intensity scale, where a higher score indicates a more pronounced perception of the corresponding attribute. Statistical analysis was based on the average of the tasters’ scores. The sensory attributes were selected to comprehensively characterize pear wines in terms of appearance, aroma, and taste.

#### 2.2.7. Entropy Weight-Technique for Order Preference by Similarity to Ideal Solution (TOPSIS) Analysis

Entropy weight-TOPSIS analysis was employed to comprehensively evaluate the effects of different enzyme concentrations on pear wine quality and to determine the optimal treatment condition within each enzyme-treatment system. Seven evaluation indicators were selected based on the physicochemical, antioxidant, color, and sensory characteristics of the pear wines, including b*, transmittance, total flavonoid content, FRAP value, fruit aroma, floral aroma, and overall balance.

First, the original evaluation matrix $R=({r_{\mathrm{ij}})}_{m\times n}$ was normalized to eliminate differences in the dimensions and magnitudes of the indicators. The entropy weight *w_j_* of each indicator was then calculated based on its information entropy:

$$p_{\mathrm{ij}}=\frac{y_{\mathrm{ij}}}{\sum_{i=1}^{m}y_{\mathrm{ij}}}$$

$$e_{j}=-\frac{1}{\ln m}\sum_{i=1}^{m}p_{\mathrm{ij}}\ln p_{\mathrm{ij}}$$

$$w_{j}=\frac{1-e_{j}}{\sum_{j=1}^{n}\left(1-e_{j}\right)}$$

where *y_ij_* is the normalized value of the *j*-th indicator for the *i*-th treatment, *e_j_* is the entropy value, and *w_j_* is the corresponding entropy weight. The weighted normalized matrix was subsequently obtained by multiplying the normalized matrix by the entropy weights. TOPSIS was then used to determine the positive ideal solution *A*^+^ and negative ideal solution *A*^−^, and the Euclidean distances $D_{i}^{+}$ and $D_{i}^{-}$ of each treatment from the two ideal solutions were calculated. The relative closeness coefficient was calculated as:

$$C_{i}=\frac{D_{i}^{-}}{D_{i}^{-}+D_{i}^{+}}$$

A higher *C_i_* value indicates better comprehensive performance. The cellulase and β-glucosidase treatment groups were evaluated separately to avoid the potential masking of enzyme-specific response patterns and to identify the optimal concentration for each enzyme.

#### 2.2.8. Data Processing and Statistical Analysis

All tests were performed in triplicate, and the results are expressed as the means ± standard deviations. The data were statistically analyzed using SPSS software (version 26.0; IBM Corporation, Armonk, NY, USA) and Origin 2018 (OriginLab Corporation, Northampton, MA, USA). The differences among experimental groups were evaluated using one-way analysis of variance (ANOVA) followed by Duncan’s multiple range test (*p* < 0.05). This post hoc test was used to assess both differences between enzyme-treated groups and the control (CK) and differences among the enzyme concentrations within each treatment system. Two-way ANOVA was performed separately for each aroma compound to evaluate the effects of enzyme type, enzyme concentration, and their interaction. The two-way model included the main effects of enzyme type and concentration and the enzyme type × concentration interaction. The control (CK) was excluded from the two-way ANOVA because it did not belong to either enzyme-treatment category. The magnitude of each effect was assessed using partial eta squared. The *p*-values obtained from the two-way ANOVA were adjusted using the Benjamini–Hochberg false discovery rate (FDR) procedure separately. The Principal Component Analysis (PCA) and Orthogonal Partial Least Squares Discriminant Analysis (OPLS-DA) were conducted using SIMCA 14.1 software (Umetrics, Malmö, Sweden). The entropy weight-TOPSIS analysis was performed using the online SPSSAU platform at https://spssau.com/ (accessed on 31 August 2026). Figures were generated using Origin 2021, R packages, and the Metware Cloud platform at https://cloud.metware.cn/#/tools/tool-list (accessed on 29 July 2026).

## 3. Results

### 3.1. Effect of Enzymatic Treatment on the Physicochemical Properties and the Antioxidant Capacity of Crown Pear Wine

Treatment with the two enzymes at different concentration gradients had a significant effect on the concentration of residual sugar in pear wine (Table 1). After enzymatic treatment, the concentration of residual sugar in pear wine increased significantly. As the concentration of cellulase added increased, the residual sugar concentration generally showed an upward trend, reaching its highest levels in Groups C4 and C5. As the concentration of β-glucosidase increased, the residual sugar concentration generally followed a pattern of first rising, then falling, and then rising again, reaching its highest level in the G4 group. After treatment with cellulase, the total acid content in pear wine decreased, but the difference was not significant (*p* > 0.05). In the pear wine treated with cellulase, the total phenolic content showed a slight but significant increase.

**Figure 2:**
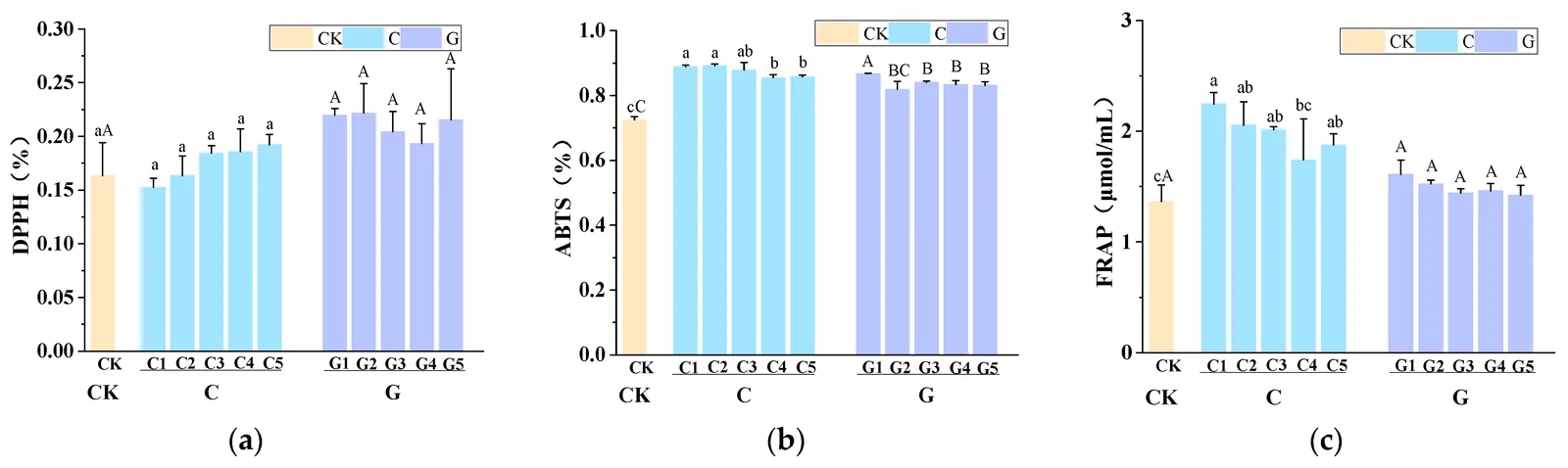
Antioxidant capacity of crown pear wine after treatment with different enzymes. (**a**) DPPH radical scavenging ability. (**b**) ABTS radical scavenging ability. (**c**) Total reducing capacity. CK, control group without enzyme treatment; C1–C5, cellulase-treated groups; G1–G5, β-glucosidase-treated groups. Different lowercase letters above the bars indicate significant differences among the cellulase-treated groups and the control group, while different uppercase letters indicate significant differences among the β-glucosidase-treated groups and the control group (*p* < 0.05).

Cellulase treatment did not significantly affect the DPPH radical scavenging rate (Figure 2a). In contrast, the ABTS radical scavenging rate of pear wines increased significantly from 72.59% (CK) to 85.50–89.20% (Figure 2b), and the total reducing capacity increased significantly from 1.36 μmol/mL (CK) to 1.73–2.25 μmol/mL (Figure 2c). Similarly, pear wines treated with β-glucosidase showed a significant increase in ABTS radical scavenging rate to 81.93–86.69% (Figure 2b).

### 3.2. Effect of Enzymatic Treatment on the Transmittance and Color of Crown Pear Wine

**Figure 3:**
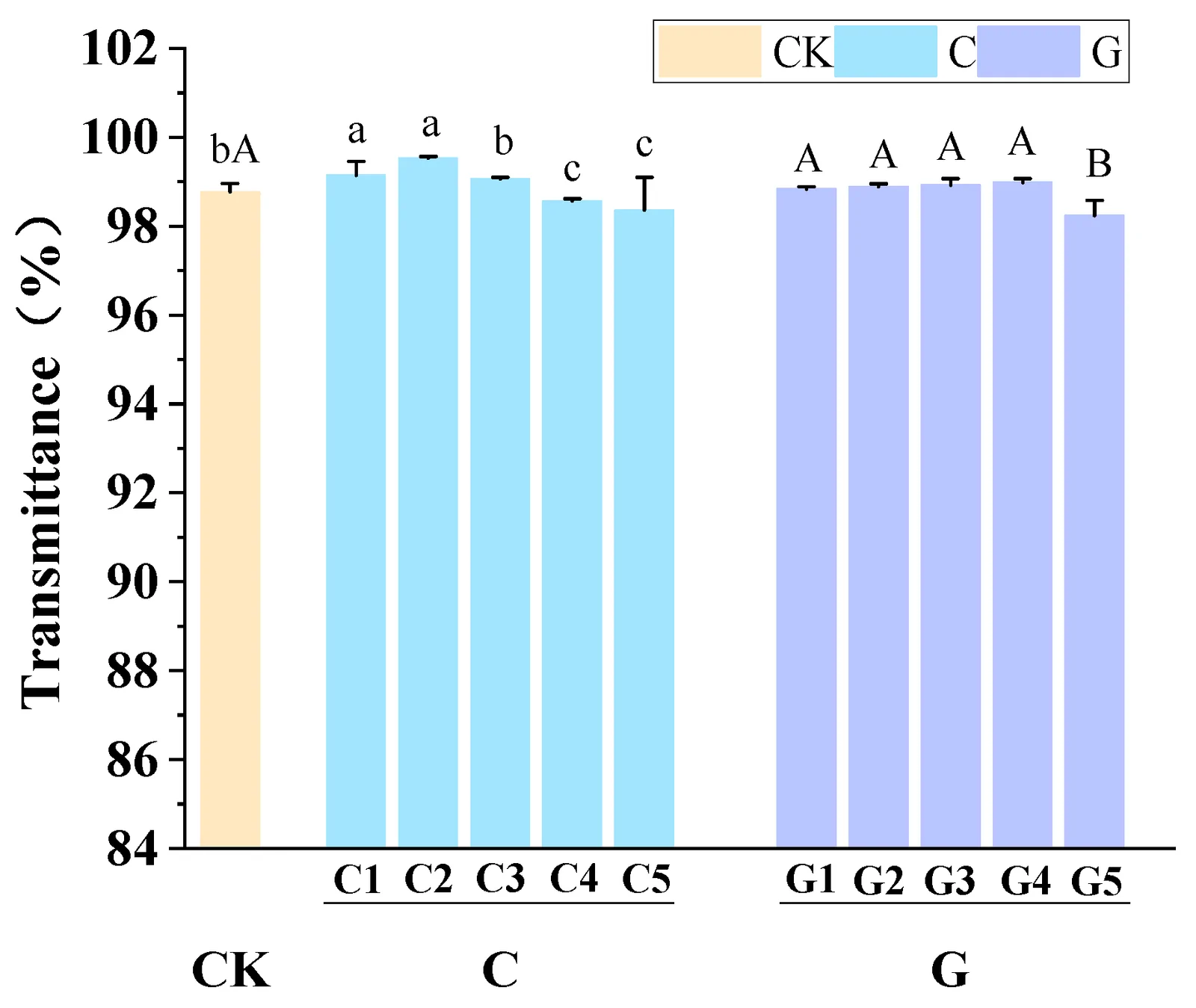
Transmittance of crown pear wine after treatment with different enzymes. CK, control group without enzyme treatment; C1–C5, cellulase-treated groups; G1–G5, β-glucosidase-treated groups. Different lowercase letters above the bars indicate significant differences among the cellulase-treated groups and the control group, while different uppercase letters indicate significant differences among the β-glucosidase-treated groups and the control group (*p* < 0.05).

As shown in Figure 3, cellulase can improve the clarity of pear wine by degrading the cellulose and hemicellulose colloids in it. The clarity of pear wine was determined using the transmittance rate. According to the experimental results, although the pear wine already had a high level of clarity (98.77%), its clarity increased significantly in Groups C1 (99.14%) and C2 (99.53%), decreased significantly in Groups C4 (98.57%) and C5 (98.37%), and showed no significant change in Groups G1–G4. However, clarity in Group G5 decreased significantly (98.24%).

Table 2 shows that an increase in cellulase concentration has a significant effect on the L* value of pear wine. Compared with the control group, the L* values in the C1 and C2 groups both increased slightly, while those in the C3, C4, and C5 groups decreased significantly. β-Glucosidase had no significant effect on the brightness of the wine. The a* values in the cellulase-treated groups were significantly lower than those in the control group, indicating that the addition of cellulase enhances the green hue of pear wine. In contrast, the a* values in the β-glucosidase-treated groups were significantly higher than those in the control group, indicating that β-glucosidase significantly reduces the green hue of pear wine. The b* values in the cellulase-treated group increased with rising enzyme concentration and were significantly higher than those in the control group, indicating that cellulase treatment significantly enhances the yellow hue of pear wine, with the yellow hue becoming more pronounced at higher concentrations. The b* values in the β-glucosidase-treated group showed no significant difference from the control group overall, with the exception of G3, suggesting that β-glucosidase does not significantly enhance the yellow hue of pear wine. In the C4 and C5 treatment groups, the color values of the pear wine increased significantly. β-glucosidase had virtually no effect on the color of the pear wine. Treatment with either enzyme had no significant effect on the hue of the pear wine.

### 3.3. Effect of Enzymatic Treatment on the Aroma Quality of Crown Pear Wine

#### 3.3.1. Analysis of the Composition of Aroma Compounds

**Figure 4:**
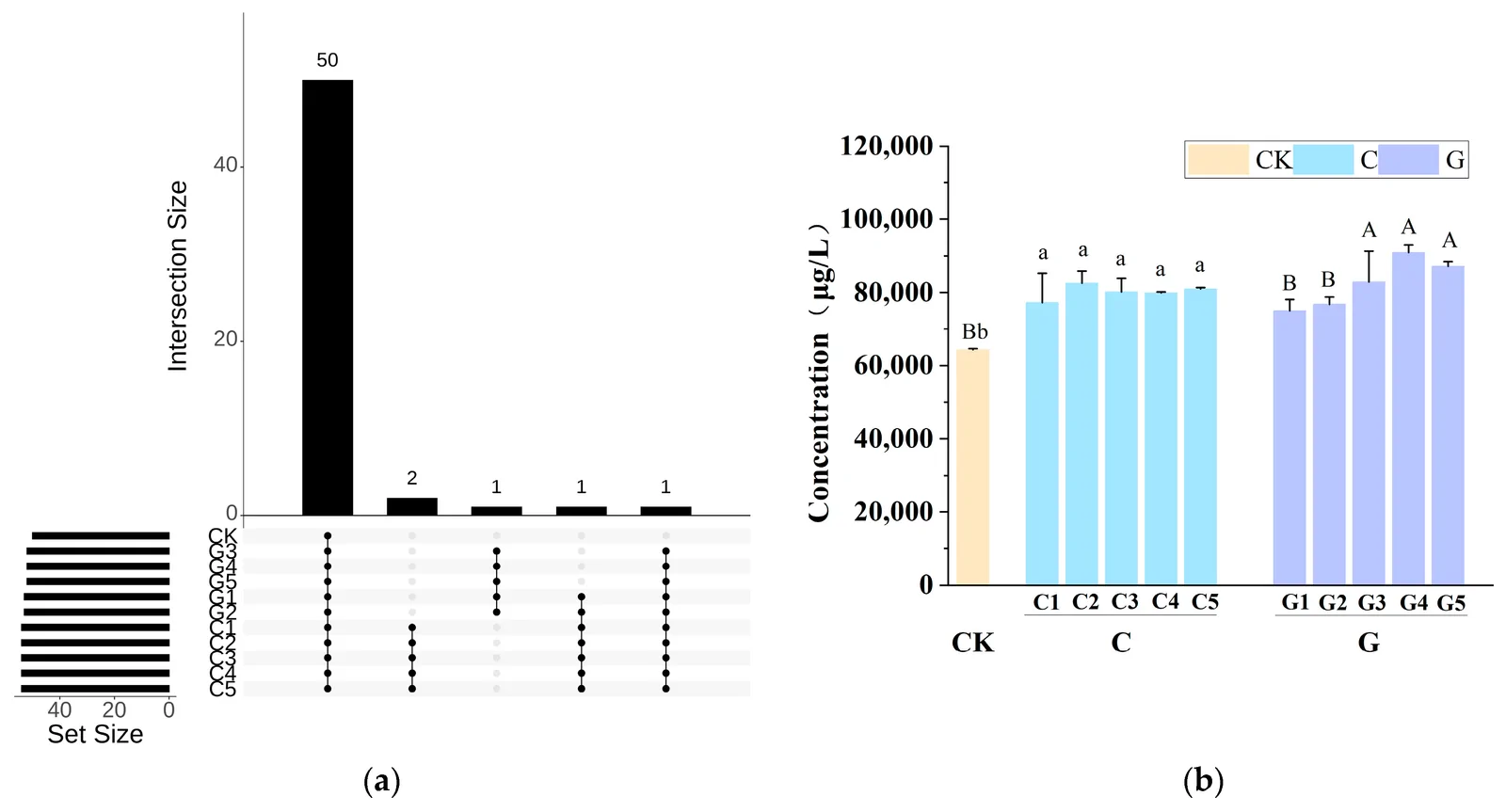
(**a**) Upset diagram of aroma compounds in pear wines. (**b**) Total aroma compound content. CK, control group without enzyme treatment; C1–C5, cellulase-treated groups; G1–G5, β-glucosidase-treated groups. Different lowercase letters above the bars indicate significant differences among the cellulase-treated groups and the control group, while different uppercase letters indicate significant differences among the β-glucosidase-treated groups and the control group (*p* < 0.05).

A total of 54 aroma compounds were detected in crown pear wine after treatment with the two enzymes. Specifically, 54 aroma compounds were detected in the treatment group with added cellulase, while 53 were detected in the treatment group with added β-glucosidase. There were differences in the types of aroma compounds before and after fermentation (Figure 4a). Four aroma compounds were detected only in the cellulase-treated pear wines, including ethyl 3-hydroxyhexanoate, limonen-6-ol pivalate, (*E*)-4-decenal, and 4-vinylphenol. Three aroma compounds were detected only in the β-glucosidase-treated pear wines, including ethyl 2,4-hexadienoate, limonen-6-ol pivalate, and (*E*)-4-decenal. There was no change in the types of aroma compounds among treatment groups using different concentration gradients of the same enzyme; however, the total amount of aroma compounds significantly increased in all enzyme-treated groups (Figure 4b), with increases ranging from 20.17% to 28.42% in the cellulase-treated group and from 16.67% to 41.32% in the β-glucosidase-treated group (percentage changes were calculated as the difference between each enzyme-treated group and CK relative to the CK value, based on the contents shown in Figure 4b). This indicates that adding enzymes before fermentation can increase the total amount of detected aroma compounds.

**Figure 5:**
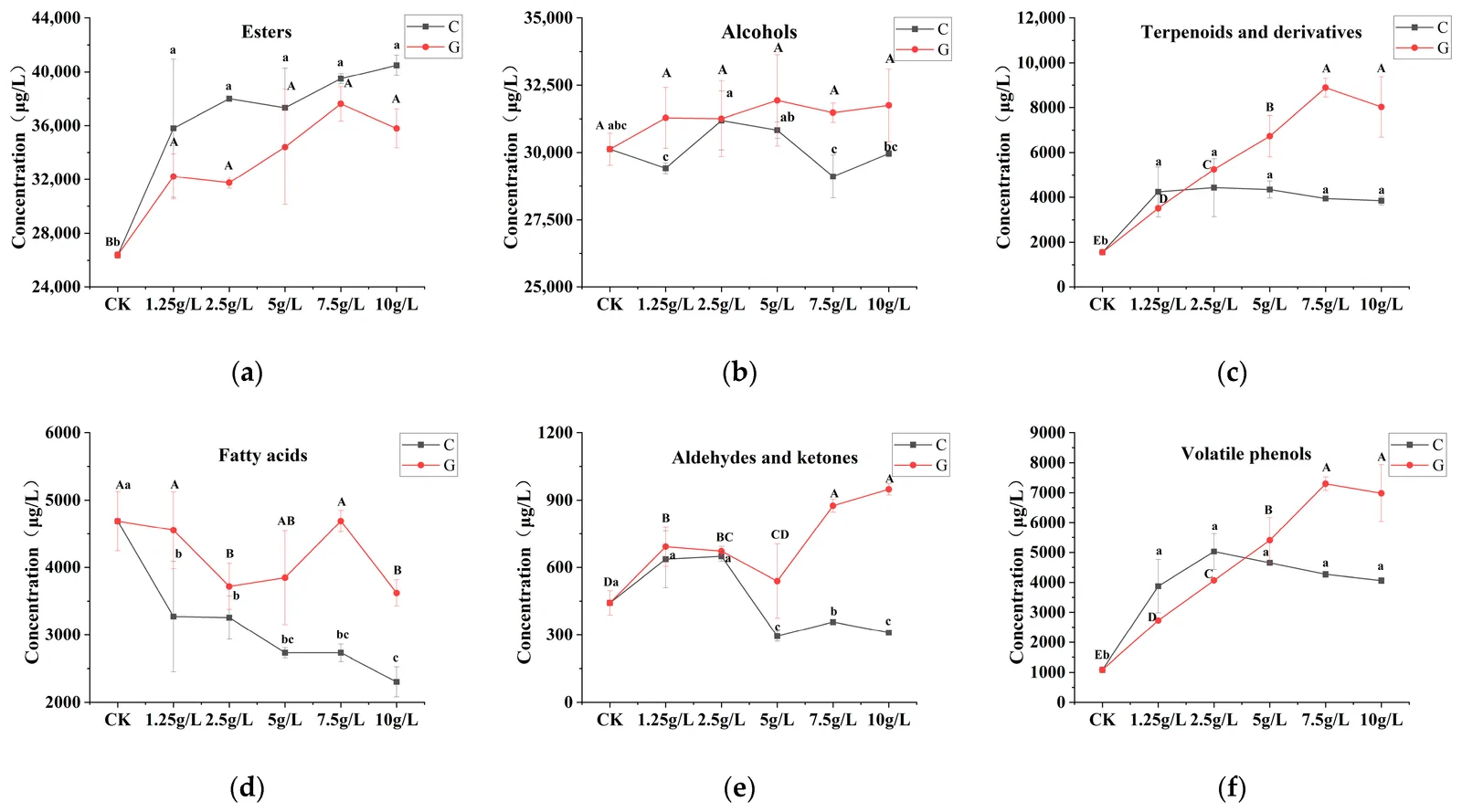
Content of various aroma compounds in crown pear wine. (**a**) Esters. (**b**) Alcohols. (**c**) Terpenoids and derivatives. (**d**) Fatty acids. (**e**) Aldehydes and ketones. (**f**) Volatile phenols. CK, control group without enzyme treatment; C, cellulase-treated groups; G, β-glucosidase-treated groups. Different lowercase letters above the data points indicate significant differences among the cellulase-treated groups and the control group, while different uppercase letters indicate significant differences among the β-glucosidase-treated groups and the control group (*p* < 0.05).

Aroma compounds were classified according to their structures, and the content of each class was calculated (Figure 5). Percentage changes were calculated as the difference between each enzyme-treated group and CK relative to the CK value, based on the contents shown in Figure 5. It was found that, compared to samples without enzyme addition, the ester content in the C1–C5 groups increased by 35.67–53.41%, and that in the G1–G5 groups increased by 20.35–42.54%; among these, the pear wine treated with cellulase had a higher overall ester content than that treated with β-glucosidase (Figure 5a). Although the ester content showed an upward trend as the enzyme concentration increased, there were no statistically significant differences among the treatment groups at different concentrations.

Alcohols are the second most abundant class of aroma compounds in pear wine and contribute to its overall aroma profile and complexity [29]. Overall, alcohol content showed a fluctuating trend across the treatment groups (Figure 5b). In groups C2 and C3, the alcohol content in the pear wines increased significantly by 8.11% and 5.75%, respectively; however, in group C4, the alcohol content decreased by 0.48% (compared to the control group). The addition of β-glucosidase had no significant effect on the alcohol content in pear wine.

Terpenes and their derivatives are primarily found in the pear. The addition of β-glucosidase caused a significant increase in the content of terpenes in pear wine; the higher the concentration of β-glucosidase added, the higher the content of terpenes (Figure 5c), with increases ranging from 126.60–473.17% in groups G1–G5. The addition of cellulase also significantly increased the content of terpenes and their derivatives in pear wine (Figure 5c), with increases ranging from 147.86–185.68% across the treatment groups; however, no significant differences were observed among the treatment groups at different concentrations. Overall, the content of these compounds in the glucosidase-treated group was much higher than in the cellulase-treated group.

Fatty acids are primarily produced during the fermentation process; when their concentration is too high, they impart an unpleasant fatty flavor to fruit wine [30]. The addition of cellulase can reduce the fatty acid content in pear wine, and as the concentration of cellulase increased, the fatty acid content continued to decrease (Figure 5d), with reductions ranging from 30.23–50.84% across the treatment groups. After adding β-glucosidase at different concentrations, the levels of fatty acid compounds in pear wine showed a fluctuating trend.

Aldehydes and ketones are primarily produced from unsaturated fatty acids under the catalysis of lipoxygenase and serve as important harmonizing components in the aroma of fruit wine [31]. Compared with samples without enzyme addition, the content of these compounds in groups G1–G5 increased significantly by 21.91–114.57%; furthermore, the higher the concentration of β-glucosidase added, the higher the content (Figure 5e). After the addition of cellulase, the content of these compounds increased by 43.89% and 46.84% in C1 and C2, and significantly decreased by 19.36–33.55% in C3–C5.

After the addition of β-glucosidase, the content of volatile phenolic compounds in pear wine increased significantly by 151.27–573.33%, and in groups G1–G4, the content continued to rise as the concentration of β-glucosidase increased (Figure 5f). The addition of cellulase caused a significant increase of 257.24–364.00% in the content of volatile phenolic compounds in pear wine, but there were no significant differences in content among the treatment groups at different concentrations.

**Figure 6:**
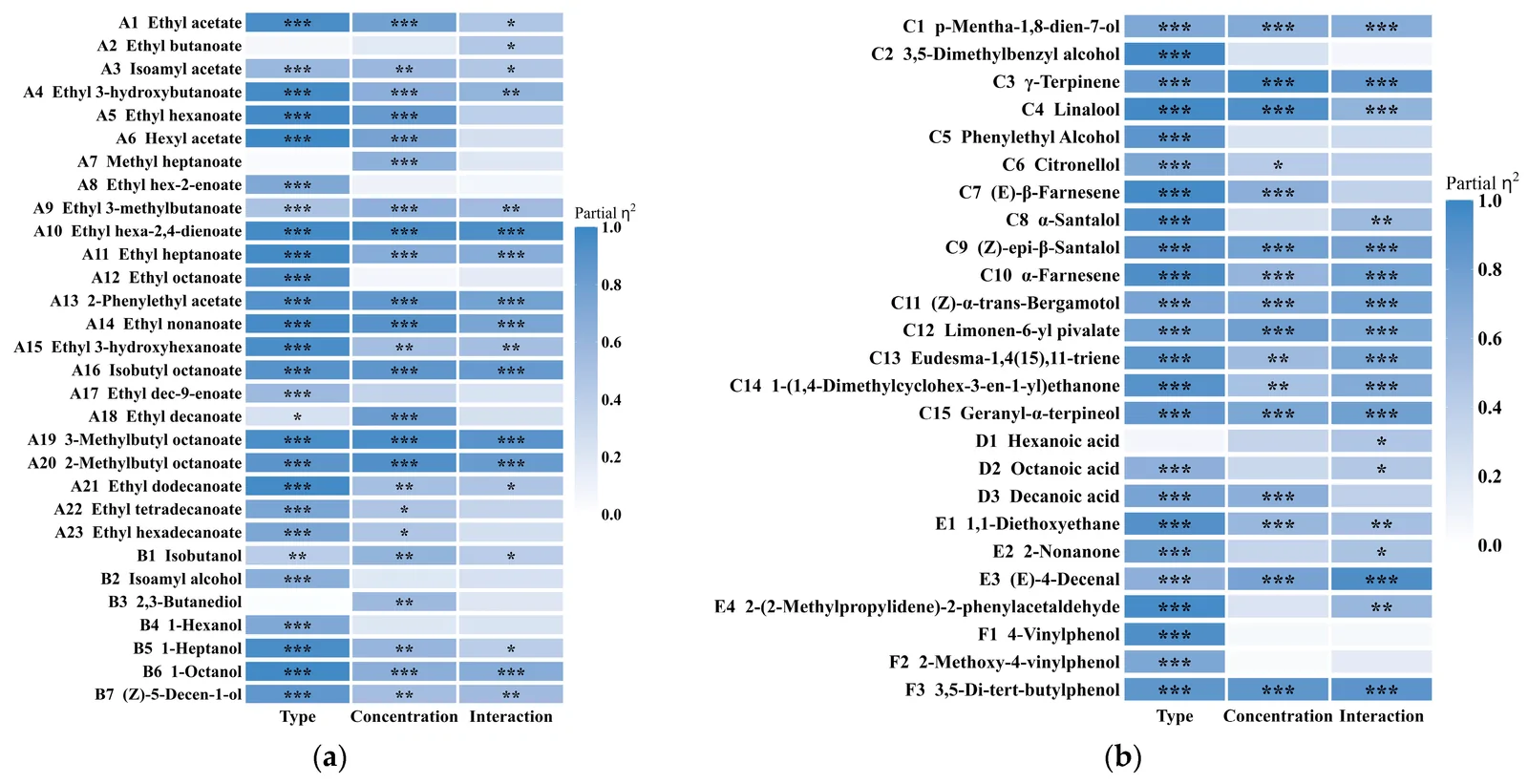
Matrix heatmap of the two-factor analysis of variance. (**a**) Esters and alcohols. (**b**) Terpenoids and derivatives, fatty acids, aldehydes and ketones, volatile phenols. * *p* < 0.05, ** *p* < 0.01, and *** *p* < 0.001.

To further clarify the effects of enzyme type and enzyme concentration on the aroma compounds in pear wine, a two-factor analysis of variance was conducted (Figure 6a,b). The results indicate that the type of enzyme had a significant effect on 93% of the aroma compounds, the enzyme concentration had a significant effect on 73% of the aroma compounds, and the interaction between the two had a significant effect on 65% of the aroma compounds. Based on the magnitude of the main effects, it can be concluded that the type of enzyme had a greater impact on the aroma compounds.

#### 3.3.2. Multivariate Statistical Analysis of Volatile Compounds

**Figure 7:**
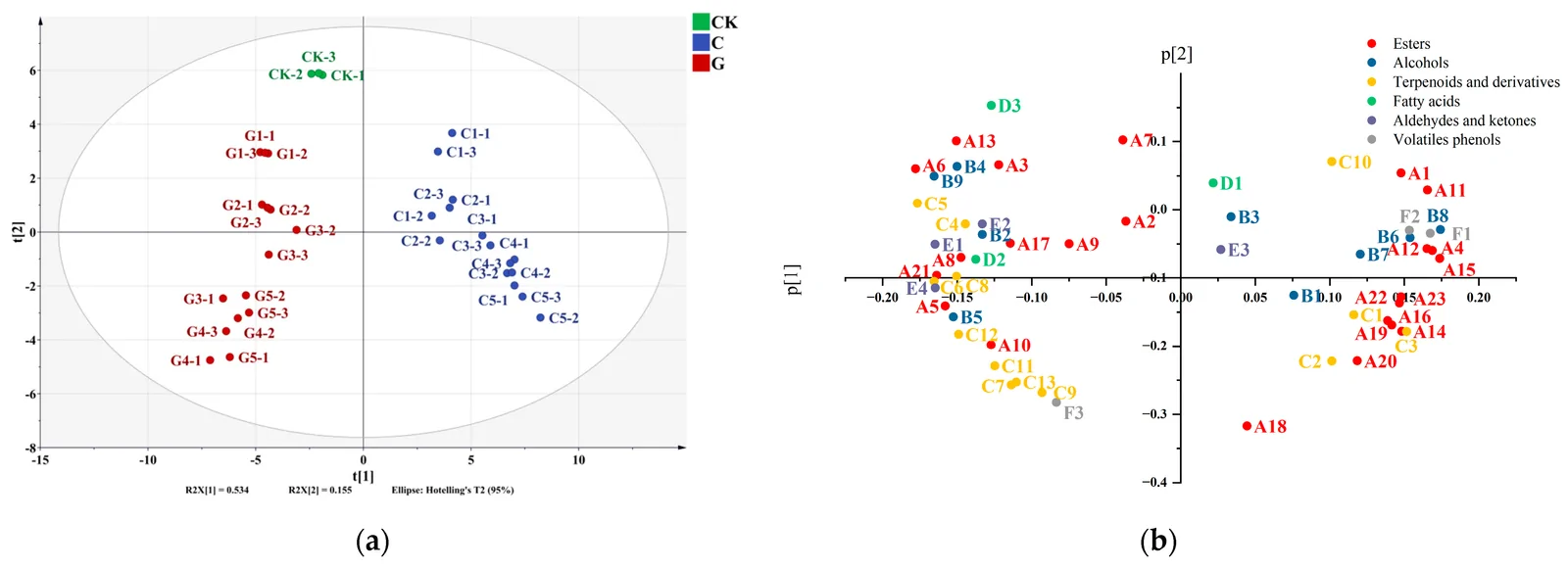
(**a**) PCA plot of aroma compounds in pear wine. (**b**) Loading plot. CK, control group without enzyme treatment; C, cellulase-treated groups; G, β-glucosidase-treated groups.

PCA was initially performed to visualize the overall variation in the volatile profiles of the wine samples. As shown in Figure 7a, PC1 and PC2 explained 53.4% and 15.5% of the total variance, respectively, accounting for 68.9% of the cumulative variance. The PCA score plot revealed different distribution patterns among the control, cellulase-treated, and β-glucosidase-treated samples. The cellulase-treated samples (C1–C5) were mainly distributed on the positive side of PC1, whereas the β-glucosidase-treated samples (G1–G5) were predominantly located on the negative side of PC1. The control sample was positioned between the two enzyme-treated groups but was clearly separated from most treated samples. These results suggested different volatile composition patterns associated with the two enzyme treatments, which were further investigated using OPLS-DA.

The PCA loading plot further illustrated the contribution and distribution of individual volatile compounds to the observed sample separation (Figure 7b). In the positive direction of the PC1 dimension, the main driving variables were 3,5-dimethylbenzyl alcohol (C2), ethyl 3-hydroxyhexanoate (A15), ethyl heptanoate (A11), ethyl 3-hydroxybutanoate (A4), ethyl octanoate (A12), 4-vinylphenol (F1), etc. The main driving variables in the negative direction were hexyl acetate (A6), (*E*)- β-farnesene (C7), phenylethyl alcohol (C5), 1,1-diethoxyethane (E1), 4-methyl-2-phenyl-2-pentenal (E4), etc.

**Figure 8:**
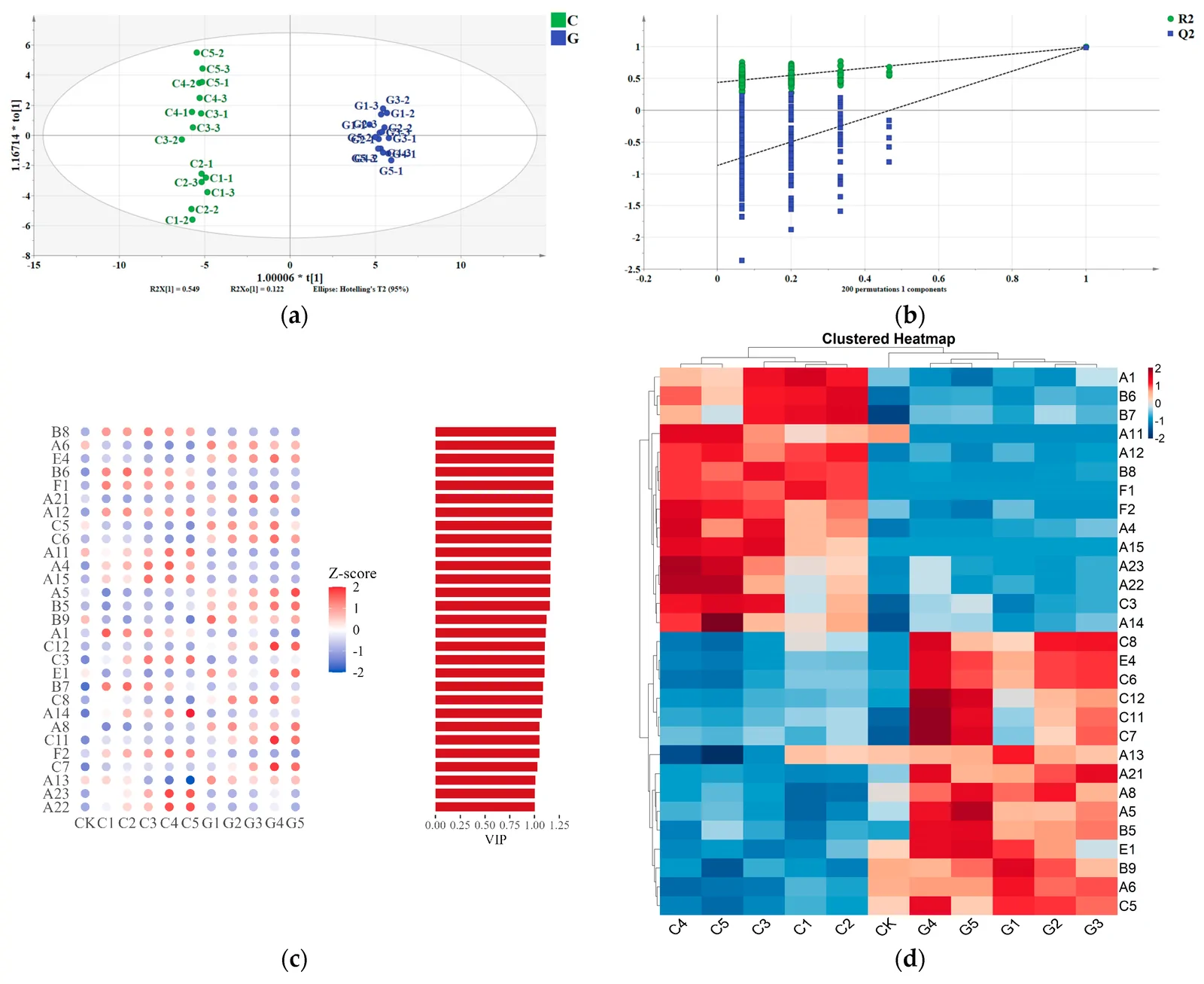
(**a**) OPLS-DA of different enzyme treatment groups. (**b**) Results of the 200 permutations test. (**c**) Heatmap of aroma compound concentrations for VIP > 1. (**d**) Clustering plot of aroma compounds for VIP > 1. CK, control group without enzyme treatment; C1–C5, cellulase-treated groups; G1–G5, β-glucosidase-treated groups.

To distinguish the effects of adding different types of enzymes on the aroma compounds of crown pear wine, OPLS-DA analysis was performed on the aroma compounds from all cellulase-treated and β-glucosidase-treated groups. The OPLS-DA model yielded R^2^X(cum) = 0.814, R^2^Y(cum) = 0.996, and Q^2^(cum) = 0.987 (Figure 8a). The model was evaluated using [systematic cross-validation] and 200 permutation tests (Figure 8b). The permutation-test regression intercepts were 0.438 for R^2^ and −0.868 for Q^2^. These results were considered together when assessing the model’s explanatory performance, predictive ability, and potential overfitting, indicating that the model was not overfitted and was valid. The results showed that crown pear wine fermented with different types of enzymes could be well distinguished using PC1 or PC2. The results can be used to analyze differences in volatile aroma compounds among pear wines subjected to different treatments.

Compounds with a Variable Importance in Projection (VIP) value greater than 1 are variables that play a key role in classification in OPLS-DA [32]. The higher the VIP value, the greater the contribution. Typically, VIP > 1 is a common criterion for screening differentially expressed metabolites, and these compounds can serve as primary biomarkers to distinguish between different treatments. An analysis of the 29 differentially expressed aroma compounds with VIP > 1 in the OPLS-DA analysis revealed that, as shown in Figure 8c, these compounds included 13 esters, 3 alcohols, 9 terpenes and their derivatives, 2 aldehydes and ketones, and 2 volatile phenols. No fatty acids were identified among the differentially expressed compounds. Therefore, enzymatic treatment primarily affects esters and terpenes.

Compared with the control group without enzyme addition, most of the differentially expressed aroma compounds showed significant differences. As shown in Figure 8c, 80% of the differentially expressed aroma compounds were present at low levels in the CK group, demonstrating that enzyme treatment can significantly increase the levels of most differentially expressed aroma compounds. Based on their trends, the differentially expressed aroma compounds can be divided into two categories: those whose levels increased significantly in the cellulase-treated group and those in the β-glucosidase-treated group (Figure 8d). In the cellulase-treated group, the compounds whose levels increased included eight esters: ethyl 3-hydroxyhexanoate, ethyl octanoate, ethyl heptanoate, ethyl 3-hydroxybutanoate, ethyl acetate, ethyl nonanoate, ethyl hexadecanoate, and ethyl tetradecanoate; the alcohols included 1-octanol and (*Z*)-5-decene-1-ol. The compounds with increased content also included 3,5-dimethylbenzyl alcohol and linalool, as well as 4-vinylphenol and 2-methoxy-4-vinylphenol.

Glucosidases release more free aroma compounds by hydrolyzing glycosylated aroma compounds [12]. In the β-glucosidase-treated group, the compounds whose levels increased included seven terpenes and their derivatives: (*E*)-β-farnesene, α-santalol, 1-(1,4-Dimethylcyclohex-3-en-1-yl) ethan-1-one, α-Farnesene, among others. In addition, there were esters: hexyl acetate, ethyl dodecanoate, ethyl hexanoate, ethyl 2-hexenoate, phenethyl acetate, and ethyl 2,4-hexadienoate. Aldehydes and ketones included 4-methyl-2-phenyl-2-pentenal and 1,1-diethoxyethane; the only alcohol present was 1-heptanol.

EOAVs were calculated for all aroma compounds (VIP > 1). According to the results (Table 3), cellulase treatment significantly increased the EOAVs of ethyl octanoate and ethyl nonanoate, while the EOAVs of ethyl acetate and ethyl heptanoate also showed increasing trends, although the differences were not statistically significant. These compounds are generally associated with fruity aroma characteristics and may contribute to the fruity aroma profile of pear wine. In addition, linalool generally imparts rose and sweet aromas, and cellulase treatment increased the EOAV of linalool in pear wine by 261.76–617.23%. Although only a minor fraction of linalool exists in glycosidically bound form, the majority is physically entrapped within the cellulose matrix [9], suggesting that cellulase-mediated hydrolysis of cellulose may liberate free linalool and thereby intensify the rose-like fragrance of the wine. Although the levels of most compounds—such as ethyl caprylate, ethyl myristate, and linalool—also increased in the β-glucosidase-treated group, the magnitude of the increase was smaller than that observed in the cellulase-treated group.

The distinctive aroma compounds in the β-glucosidase-treated group were primarily terpenes and their derivatives as well as esters, which impart floral and sweet aromas, such as ethyl dodecanoate, (*E*)-β-farnesene, α-Farnesene, and 2-phenethyl acetate. These compounds increased by up to 166.61%, 47.93%, 277.82%, and 18.79%, respectively at their maximum levels. In addition, there were compounds such as hexyl acetate and 1,1-diethoxyethane, which have been reported to exhibit fruity and green odor characteristics [19,28]. Most of these compounds reached higher EOAVs at G4.

#### 3.3.3. Sensory Analysis Results

**Figure 9:**
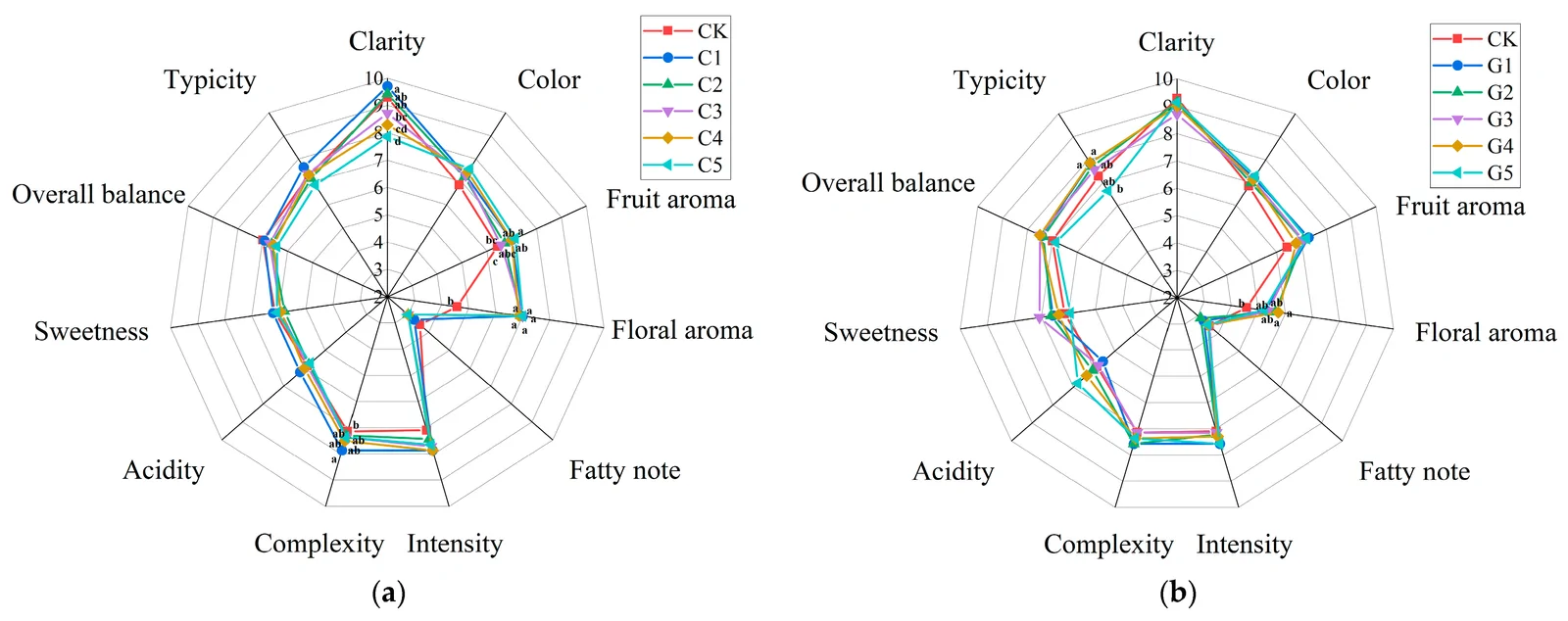
Radar map of sensory scores o crown pear wine after treatment with different enzymes. (**a**) Cellulase-treated group. (**b**) β-Glucosidase-treated group. CK, control group without enzyme treatment; C1–C5, cellulase-treated groups; G1–G5, β-glucosidase-treated groups. Different lowercase letters indicate significant differences among the corresponding treatment groups and CK within each panel (*p* < 0.05).

Based on the results of the sensory analysis (Figure 9), the addition of cellulase enhanced the floral aroma of the wine. At the same time, the addition of cellulase enhanced the fruity aroma. When the cellulase concentration was increased to 5 g/L–10 g/L, the clarity of the pear wine decreased. Based on the sensory evaluation results, C1 received higher ratings in complexity.

After the addition of β-glucosidase, floral aroma was also enhanced, likely due to a significant increase in terpenoid content, which rose by 42.63–140.90% across the treatment groups. The fruit aroma of the wine was slightly enhanced, but there was no significant difference. Typicity of some treatment groups was higher, but the overall balance, complexity, and typicity scores of most groups were not particularly outstanding. To further evaluate the comprehensive performance of different concentrations for each enzyme, an entropy-weighted TOPSIS analysis was conducted.

#### 3.3.4. Correlation Analysis of Quality Attributes

**Figure 10:**
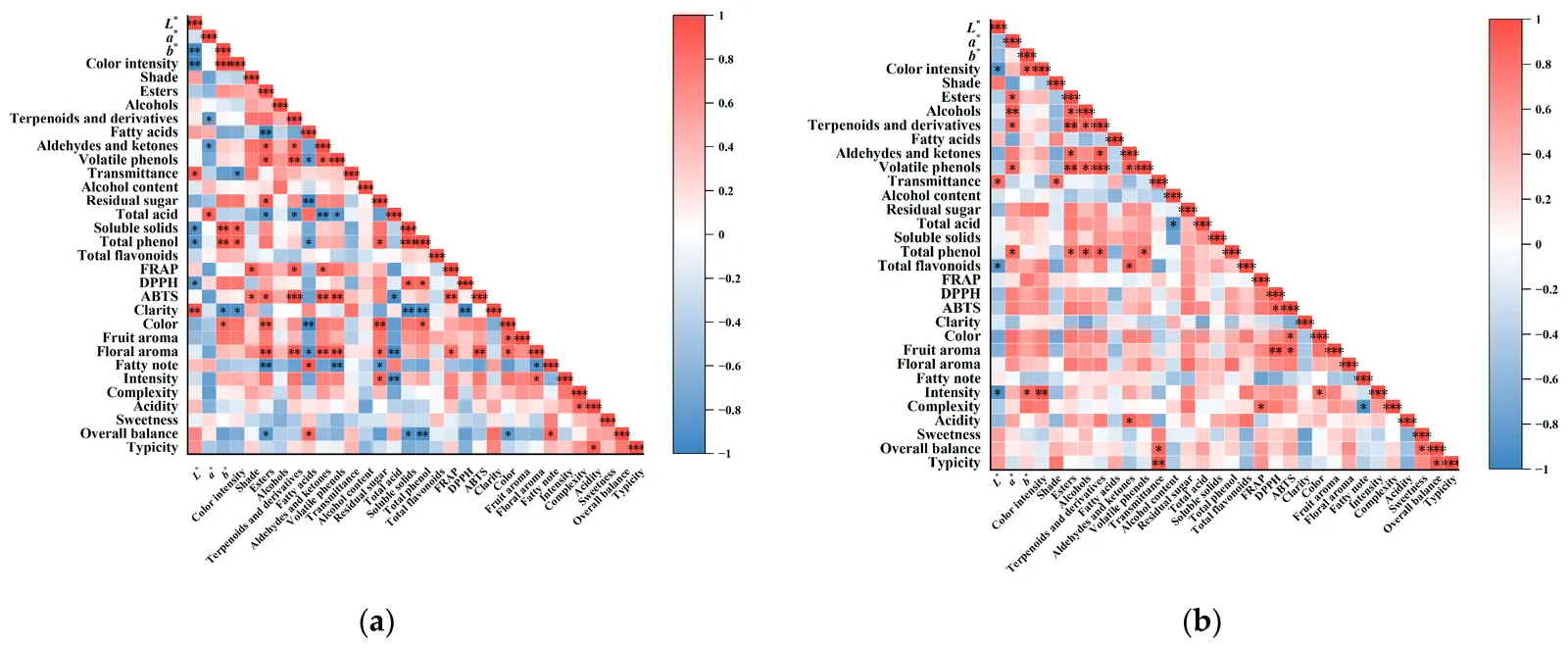
Heat map of correlation analysis between various quality indicators and sensory evaluation of pear wine. (**a**) Cellulase-treated group. (**b**) β-Glucosidase-treated group. * *p* < 0.05, ** *p* < 0.01, and *** *p* < 0.001.

Before the comprehensive evaluation of the enzyme-treated wines, Pearson correlation analysis was performed to examine the relationships among the measured physicochemical, antioxidant, color, and sensory attributes (Figure 10). Because cellulase and β-glucosidase may exert different effects on the formation and release of volatile compounds, the correlation structures among volatile and sensory attributes may also be enzyme-dependent. For example, linalool (C4), an aroma compound associated with floral odor characteristics [27], exhibited different response patterns between the two enzyme-treatment systems, as reflected by its EOAVs (Table 3). These differences suggest that the relationships among aroma compounds and sensory attributes may vary among different enzymes. Therefore, Pearson correlation analysis was conducted separately for the cellulase- and β-glucosidase-treated samples rather than pooling the two treatment systems. This approach enabled the enzyme-specific relationships among quality attributes to be characterized and minimized the potential masking of treatment-specific correlation patterns. Several quality attributes exhibited relatively strong correlations within each enzyme-treatment system, suggesting potential information redundancy among some evaluation indicators. Accordingly, the correlation analysis was used as an auxiliary criterion for selecting representative indicators for subsequent comprehensive evaluation, while ensuring that the major quality dimensions of the wine.

#### 3.3.5. Entropy Weight-TOPSIS Comprehensive Evaluation

In the Pearson correlation analysis results, indicator pairs showing strong correlations (|r| ≥ 0.8) were considered potentially redundant and were further evaluated in combination with their biological relevance, variation characteristics, and the objectives of the cellulase and β-glucosidase treatments.

Seven indicators representing complementary aspects of wine quality were selected for the entropy weight–TOPSIS evaluation. Among the seven selected indicators, b* was classified as an undesirable indicator. Under this evaluation framework, lower b* values are preferred in order to preserve the relatively light color of crown pear wine and prevent excessive oxidation and yellowing. Transmittance, total flavonoid content, FRAP, fruity aroma, floral aroma, and overall balance were classified as beneficial indicators. These indicators collectively represented appearance, clarification, antioxidant properties, and sensory characteristics, while minimizing potential information redundancy among highly correlated variables. The entropy-derived weights for the seven indicators were calculated separately; the corresponding values are shown in Table 4 and Table 5.

In the cellulase-treated group, the entropy weight analysis showed that the contribution of each indicator to the comprehensive evaluation followed the order: Transmittance > Fruit aroma > Overall balance > b* > FRAP > Total flavonoids > Floral aroma. While in the β-Glucosidase-treated group, the order was: Total flavonoids > FRAP > Overall balance > b* > Fruit aroma > Floral aroma > Transmittance.

In the cellulase-treated group, the relative proximity coefficients for C1, C2, and C3 were all higher than those in the control group, with C1 having the highest coefficient, while the values for C4 and C5 were slightly lower (Table 6). In contrast, all samples treated with β-glucosidase had higher relative proximity coefficients than the control group. Among them, G5 had the highest coefficient (Table 7). Furthermore, under the weight perturbation scenario, C1 and G5 still ranked highest in the cellulase and β-glucosidase systems, respectively; therefore, the entropy-weighted TOPSIS results demonstrate robustness.

## 4. Discussion

Enzymatic treatment altered the physicochemical, antioxidant, clarification, color, and aroma characteristics of pear wine, but the magnitude and direction of these effects depended strongly on enzyme type and concentration.

Regarding the physicochemical properties, cellulase treatment significantly affected several parameters, including residual sugar, total acidity, soluble solids, and total phenolic content, whereas β-glucosidase treatment mainly affected residual sugar content (Table 1). This may be because, as the amount of cellulase increased, the cellulose in the cells was enzymatically hydrolyzed into glucose, cellobiose, and cellulose oligosaccharides [16], and β-glucosidases can cleave the glycosidic bonds of bound glycosides, releasing free monosaccharides [12]. After adding cellulase, the total phenolic content in the pear wine increased significantly, possibly because cellulase promotes the rupture of cell walls, releasing phenolic compounds.

Both ABTS and FRAP results indicated that treatment with the two enzymes enhanced the antioxidant capacity of pear wine, possibly because cellulase disrupted the macromolecular structure of the cells, promoting the release of more antioxidant substances from the cells [16]. β-Glucosidase may also influence antioxidant properties through the hydrolysis of certain glycosylated phenolic compounds [33].

Moderate cellulase treatment improved transmittance, whereas excessive cellulase addition was associated with reduced transmittance and lower L* values. The increased color intensity observed at C4 and C5 may further indicate a tendency toward oxidative browning. The reduced clarity at higher cellulase concentrations may be related to changes in the composition and interactions of colloidal components, such as polysaccharides and proteins, which can influence the stability and transmittance of wine [34,35]. However, the specific mechanisms underlying these changes remain unclear.

Several aroma compounds were detected only in the enzymatic treatment, suggesting that cellulase and β-glucosidase may have influenced the availability and subsequent transformation of aroma-related precursors. In the cellulase-treated samples, ethyl 3-hydroxyhexanoate and 4-vinylphenol were detected only after enzymatic treatment. These compounds have been associated with fruity and phenolic aroma characteristics, respectively, while 4-vinylphenol brings medicinal, spicy or smoky flavors at a high concentration [36,37]. However, Limonen-6-ol pivalate was not detected in the high-concentration β-glucosidase treatments (G3–G5), which may be related to changes in precursor availability or subsequent transformation of the compound at higher enzyme concentrations. The specific mechanism requires further investigation. Ethyl 2,4-hexadienoate was detected only in the β-glucosidase-treated wines under the analytical conditions employed. This compound has been reported to exhibit fruity, sweet, and pineapple odor characteristics [38]. Overall, these results suggest that cellulase and β-glucosidase shared some effects on volatile precursor transformation but differed in the composition and magnitude of their aroma responses.

Both enzyme treatments altered the content of aroma compounds in pear wine, but their mechanisms of action may differ to some extent. Cellulase treatment disrupted the fruit cell-wall structure and facilitated the release of intracellular and cell-wall-associated aroma precursors [8]. Therefore, the greater increase in ester content observed following cellulase treatment may be associated with changes in the availability of aroma-related compounds. β-glucosidase can hydrolyze glycosidically bound aroma precursors, potentially facilitating the release of free terpenoid compounds. This mechanism may partially explain the greater increase in terpenoids observed in the β-glucosidase-treated samples. Esters generally impart a pleasant fruity aroma to fruit wines and are important aroma compounds in such beverages. Therefore, the increased ester abundance after enzyme treatment may contribute to the fruity aroma characteristics of pear wine. Terpenes and their derivatives are important contributors to floral aroma in fruit wines. Their increased abundance may explain the differences in floral aroma perception among treatments. This interpretation was further supported by the sensory evaluation results.

The effects of the two enzymes on aldehydes and ketones were different. Cellulase treatment increased their levels at lower concentrations but decreased them at higher concentrations, whereas β-glucosidase treatment consistently increased their levels. These differences may be associated with enzyme-dependent changes in the availability and transformation of aroma-related precursors during fermentation [7]. In fruit wines, some volatile phenolic compounds are present as odorless glycosidically bound precursors, which may release aroma-active compounds upon hydrolysis. β-Glucosidase may facilitate the release of free volatile phenolic compounds by hydrolyzing glycosidic bonds [39]. This might be related to the continuous increase in the content of volatile phenolic substances in the β-glucosidase treatment group. However, the specific biochemical pathways responsible for these changes require further investigation.

In the cellulase-treated group, the fatty acid content decreased significantly, whereas the β-glucosidase-treated group showed a fluctuating response. These differences may be associated with the distinct effects of the two enzymes on the transformation of aroma-related precursors during fermentation. For example, as cellulase concentration increases, cellulose is degraded into smaller molecules; the increase in fermentation substrates stimulates yeast proliferation and metabolism within the fermentation system, thereby promoting alcohol production while controlling the formation of fatty acid compounds [40]. The metabolic pathways responsible for the changes require further investigation.

The enzyme type affected a larger proportion of aroma compounds than enzyme concentration, suggesting that enzyme type played a major role in determining the volatile profile. This was further supported by the PCA results.

To further investigate the differences in the effects of various types of enzymes on the aroma compounds in pear wine, an OPLS-DA analysis was conducted. The results showed that treatment with different enzymes primarily led to differences in the types and concentrations of esters and terpenes.

Through cluster analysis of aroma compounds with VIP > 1 and calculation of EOAVs, it was found that cellulase treatment can impart a more intense fruity aroma to pear wine by increasing the content of esters such as ethyl octanoate, ethyl hexanoate, and ethyl nonanoate, while simultaneously enhancing the intensity of the rose aroma by increasing the content of linalool. β-glucosidase enhanced the levels of terpenes and their derivatives, as well as some esters and aldehydes, thereby imparting floral, fruity, and refreshing aromas to the pear wine.

Overall, the two enzyme treatments exhibited distinct effects on the quality characteristics of pear wine. Cellulase treatment appeared to exert a broader effect on the fruit matrix by disrupting cell-wall structures and facilitating the release of intracellular and cell-wall-associated components. This effect was reflected not only in the increased accumulation of volatile esters and enhanced fruity aroma, but also in the marked changes in transmittance and color characteristics. In contrast, β-glucosidase treatment showed a more pronounced effect on terpenes and their derivatives, consistent with its ability to hydrolyze glycosidically bound aroma precursors and release free volatile compounds. Therefore, cellulase treatment tended to produce a more fruity and clarification-oriented quality profile, whereas β-glucosidase treatment produced a more aroma-release-oriented profile characterized by stronger floral and terpene-related characteristics.

The results of the sensory quantitative descriptive analysis showed that the addition of cellulase enhanced the floral aroma of pear wine. This may be due to the significant increase in linalool content in the cellulase-treated groups; the threshold for linalool is relatively low, and the rose aroma in the treated groups was more easily perceived, imparting a distinct floral aroma to the pear wine [41]. The intensification of the fruity aroma may be due to an increase in the content of esters. However, when the concentration of cellulase added was too high, the clarity of the pear wine decreased, which was consistent with the transmittance results measured earlier; this may affect the evaluators’ assessment of the pear wine. β-Glucosidase treatment, in contrast, produced a more pronounced enhancement of floral aroma intensity, but an improvement in aroma does not necessarily mean a corresponding increase in the overall rating. These findings further suggested that the two enzymes may produce different sensory profiles. Therefore, further comprehensive evaluation is needed.

Seven indicators were selected for the entropy-weighted TOPSIS analysis through Pearson correlation analysis. The entropy-weighted TOPSIS analysis provided an integrated assessment of the multiple quality attributes affected by enzymatic treatment. For cellulase, C1 showed the highest comprehensive score among the tested concentrations, indicating that a relatively low enzyme concentration provided the most favorable balance among clarification, aroma, color, and antioxidant-related attributes. The decline in comprehensive performance at higher cellulase concentrations suggests that further increases in enzyme dosage may not translate into overall quality improvement despite changes in individual aroma or color parameters. In contrast, G5 showed the highest comprehensive performance within the β-glucosidase treatment series, indicating that the stronger aroma-release and antioxidant-related responses at higher β-glucosidase concentrations outweighed the relatively limited improvement in transmittance. These results highlight that the most suitable enzyme concentration within the tested range depends on the quality attributes emphasized by each enzyme system rather than on aroma intensity alone. The differences suggest that the two enzymes may be used for different quality-oriented purposes in pear wine production. It should be noted that the independently calculated TOPSIS scores were used only to rank concentrations within each enzyme-treatment system and were not interpreted as a direct measure of the relative superiority of the two enzymes.

Despite these findings, several limitations must be acknowledged. First, although the roles of cellulase and β-glucosidase in cell wall degradation and the hydrolysis of aroma precursors bound to glycosidic bonds were discussed, these mechanisms were not directly verified through microstructural or precursor-level analyses. Second, the sensory evaluation employed a quantitative descriptive analysis method, and no consumer acceptance tests were conducted. Finally, from a practical perspective, although 10 g/L β-glucosidase was the highest-ranked treatment within the tested concentrations, the relatively high enzyme dosage may increase production costs. As no economic assessment was conducted in this study, it is not yet known whether the quality improvements achieved can offset the increased costs and processing requirements. Future research should integrate microstructural characterization, metabolite analysis, and consumer sensory evaluation to further validate the mechanisms by which enzymatic treatment affects the components in pear wine, the practical applicability of these treatments, particularly at higher enzyme concentrations, requires further evaluation considering their economic feasibility.

## 5. Conclusions

This study demonstrated that enzymatic treatment effectively modulated the measured quality attributes of pear wines, including physicochemical properties, clarity, color, antioxidant capacity, aroma composition, and sensory characteristics, with the responses varying according to enzyme type and concentration. Cellulase treatment resulted in greater increases in ester content and improved transmittance at lower concentrations, whereas β-glucosidase treatment produced larger increases in terpenes and their derivatives. Entropy-weighted TOPSIS identified 1.25 g/L cellulase and 10 g/L β-glucosidase as the highest-ranked treatments among the tested concentrations within their respective enzyme-treatment systems. Overall, the results indicate that the two enzymes provide complementary strategies for regulating pear wine quality and aroma.

## Figures and Tables

**Table 1:** Physicochemical properties of crown pear wine after treatment with different enzymes.

	CK	C1	C2	C3	C4	C5	G1	G2	G3	G4	G5
Alcohol content(%)	8.44 ± 0.66 ^aA^	8.23 ± 0.01 ^a^	8.85 ± 0.70 ^a^	8.95 ± 1.07 ^a^	7.90 ± 1.05 ^a^	9.14 ± 0.64 ^a^	8.35 ± 0.42 ^A^	8.61 ± 0.78 ^A^	8.33 ± 0.03 ^A^	8.14 ± 0.07 ^A^	8.49 ± 0.77 ^A^
Residual sugar (g/L)	0.59 ± 0.09 ^dB^	0.84 ± 0.00 ^bc^	0.78 ± 0.00 ^c^	0.91 ± 0.00 ^ab^	1.00 ± 0.04 ^a^	1.00 ± 0.04 ^a^	0.94 ± 0.13 ^A^	0.94 ± 0.04 ^A^	0.72 ± 0.09 ^B^	1.00 ± 0.04 ^A^	0.94 ± 0.04 ^A^
Total acid (g/L)	2.74 ± 0.07 ^aA^	2.32 ± 0.13 ^b^	2.41 ± 0.00 ^ab^	2.44 ± 0.04 ^ab^	2.33 ± 0.29 ^b^	2.41 ± 0.05 ^ab^	2.97 ± 0.13 ^A^	2.72 ± 0.09 ^A^	2.99 ± 0.03 ^A^	3.07 ± 0.00 ^A^	2.88 ± 0.27 ^A^
Soluble solids (°Brix)	8.20 ± 0.14 ^cA^	8.15 ± 0.07 ^c^	8.30 ± 0.14 ^bc^	8.35 ± 0.07 ^abc^	8.65 ± 0.21 ^ab^	8.70 ± 0.14 ^a^	8.20 ± 0.28 ^A^	8.10 ± 0.14 ^A^	8.15 ± 0.07 ^A^	8.65 ± 0.07 ^A^	8.30 ± 0.28 ^A^
Total phenol (mg GAE/mL)	0.18 ± 0.01 ^cA^	0.19 ± 0.00 ^c^	0.20 ± 0.01 ^bc^	0.21 ± 0.03 ^abc^	0.24 ± 0.03 ^ab^	0.25 ± 0.00 ^a^	0.19 ± 0.02 ^A^	0.20 ± 0.01 ^A^	0.20 ± 0.02 ^A^	0.20 ± 0.00 ^A^	0.20 ± 0.01 ^A^
Total flavonoids (mg RE/mL)	0.11 ± 0.00 ^aA^	0.11 ± 0.00 ^a^	0.11 ± 0.00 ^a^	0.09 ± 0.00 ^b^	0.11 ± 0.00 ^a^	0.12 ± 0.01 ^a^	0.12 ± 0.00 ^A^	0.11 ± 0.00 ^A^	0.11 ± 0.01 ^A^	0.13 ± 0.01 ^A^	0.14 ± 0.00 ^A^

Note: Data are expressed as mean ± standard deviation. Different superscript letters in the same row indicate significant differences (*p* < 0.05). Lowercase letters indicate a significant difference between the cellulase-treated group and the control group. Uppercase letters indicate a significant difference between the β-glucosidase-treated group and the control group. CK, control group without enzyme treatment; C1–C5, cellulase-treated groups; G1–G5, β-glucosidase-treated groups.

**Table 2:** Color parameters of crown pear wine after treatment with different enzymes.

	CK	C1	C2	C3	C4	C5	G1	G2	G3	G4	G5
L*	98.44 ± 0.07 ^aA^	98.65 ± 0.27 ^a^	98.80 ± 0.02 ^a^	98.30 ± 0.02 ^b^	97.90 ± 0.09 ^b^	97.43 ± 0.44 ^b^	98.25 ± 0.07 ^A^	98.36 ± 0.02 ^A^	98.43 ± 0.05 ^A^	98.37 ± 0.04 ^A^	98.08 ± 0.25 ^A^
a*	−0.14 ± 0.04 ^aD^	−0.34 ± 0.07 ^c^	−0.29 ± 0.02 ^bc^	−0.24 ± 0.07 ^bc^	−0.31 ± 0.05 ^bc^	−0.21 ± 0.03 ^ab^	−0.07 ± 0.02 ^C^	−0.05 ± 0.01 ^BC^	−0.02 ± 0.02 ^AB^	−0.05 ± 0.02 ^BC^	0.00 ± 0.02 ^A^
b*	4.08 ± 0.21 ^bCD^	4.40 ± 0.64 ^a^	4.12 ± 0.16 ^a^	4.40 ± 0.30 ^a^	5.48 ± 0.06 ^a^	6.16 ± 0.38 ^a^	4.75 ± 0.09 ^A^	4.29 ± 0.04 ^BC^	4.01 ± 0.08 ^D^	4.47 ± 0.02 ^B^	4.40 ± 0.22 ^B^
Color intensity	0.092 ± 0.00 ^bC^	0.097 ± 0.01 ^b^	0.083 ± 0.00 ^b^	0.100 ± 0.00 ^b^	0.128 ± 0.00 ^a^	0.146 ± 0.02 ^a^	0.105 ± 0.00 ^AB^	0.097 ± 0.00 ^C^	0.091 ± 0.00 ^C^	0.098 ± 0.00 ^BC^	0.109 ± 0.01 ^A^
Shade	2.92 ± 0.16 ^aA^	3.52 ± 0.06 ^a^	3.59 ± 0.06 ^a^	3.25 ± 0.08 ^a^	3.14 ± 0.10 ^a^	3.15 ± 0.31 ^a^	2.87 ± 0.13 ^A^	2.90 ± 0.03 ^A^	2.81 ± 0.10 ^A^	2.90 ± 0.05 ^A^	2.69 ± 0.11 ^A^

Note: Data are expressed as mean ± standard deviation. Different superscript letters in the same row indicate significant differences (*p* < 0.05). Lowercase letters indicate a significant difference between the cellulase-treated group and the control group. Uppercase letters indicate a significant difference between the β-glucosidase-treated group and the control group. CK, control group without enzyme treatment; C1–C5, cellulase-treated groups; G1–G5, β-glucosidase-treated groups.

**Table 3:** Estimated odor activity values of the main differential aroma compounds in crown pear wines.

Number	Name	Threshold (μg/L)	Aroma Description	EOAV										
				CK	C1	C2	C3	C4	C5	G1	G2	G3	G4	G5
B8	3,5-dimethylbenzyl alcohol	n.f	n.f	n.f	n.f	n.f	n.f	n.f	n.f	n.f	n.f	n.f	n.f	n.f
A15	Ethyl 3-hydroxyhexanoate	n.f	n.f	n.f	n.f	n.f	n.f	n.f	n.f	n.f	n.f	n.f	n.f	n.f
B6	1-Octanol	120	Citrus, rose smell, fatty [25]	0.75 ± 0.07 ^dA^	2.37 ± 0.06 ^ab^	2.53 ± 0.16 ^a^	2.31 ± 0.16 ^b^	2.17 ± 0.09 ^b^	1.79 ± 0.10 ^c^	0.94 ± 0.18 ^A^	1.16 ± 0.09 ^A^	1.07 ± 0.00 ^A^	1.08 ± 0.03 ^A^	1.11 ± 0.10 ^A^
F1	4-Vinylphenol	180	Phenolic, medicinal [20]	n.d	3.11 ± 0.90	2.72 ± 1.10	2.61 ± 0.16	2.79 ± 0.44	2.73 ± 0.05	n.d	n.d	n.d	n.d	n.d
A12	Ethyl octanoate	5	Pineapple, pear, floral [21]	2772.38 ± 58.96 ^bA^	4526.41 ± 644.22 ^a^	4609.08 ± 67.17 ^a^	4354.92 ± 334.72 ^a^	4567.43 ± 4.99 ^a^	4695.03 ± 38.76 ^a^	3133.48 ± 173.31 ^A^	2902.09 ± 124.76 ^A^	2979.26 ± 377.58 ^A^	3093.30 ± 85.68 ^A^	2811.71 ± 174.72 ^A^
A11	Ethyl heptanoate	2	Berry, plum [21]	9.96 ± 0.79 ^a^	6.48 ± 1.48 ^a^	8.79 ± 0.33 ^a^	10.28 ± 0.03 ^a^	14.16 ± 2.06 ^a^	14.28 ± 3.04 ^a^	n.d	n.d	n.d	n.d	n.d
A4	Ethyl 3-hydroxybutanoate	20,000	Fruity [23]	0.001 ± 0.00 ^cA^	0.002 ± 0.00 ^b^	0.002 ± 0.00 ^b^	0.002 ± 0.00 ^a^	0.002 ± 0.00 ^a^	0.002 ± 0.00 ^b^	0.001 ± 0.00 ^A^	0.001 ± 0.00 ^A^	0.001 ± 0.00 ^A^	0.001 ± 0.00 ^A^	0.001 ± 0.00 ^A^
A1	Ethyl acetate	7500	Fruity, sweet [21]	0.09 ± 0.02 ^aA^	0.14 ± 0.02 ^a^	0.13 ± 0.01 ^a^	0.13 ± 0.00 ^a^	0.12 ± 0.01 ^a^	0.11 ± 0.00 ^a^	0.09 ± 0.00 ^A^	0.08 ± 0.00 ^A^	0.10 ± 0.00 ^A^	0.08 ± 0.00 ^A^	0.08 ± 0.00 ^A^
C3	Linalool	10	Citrus, orange, floral [27]	11.95 ± 0.01 ^dA^	43.23 ± 5.98 ^c^	63.74 ± 6.09 ^b^	82.94 ± 7.77 ^a^	79.90 ± 5.68 ^a^	85.71 ± 0.74 ^a^	18.06 ± 0.43 ^A^	26.47 ± 0.20 ^A^	29.16 ± 4.44 ^A^	38.31 ± 0.19 ^A^	44.19 ± 0.03 ^A^
B7	(*Z*)-5-Decen-1-ol	n.f	n.f	n.f	n.f	n.f	n.f	n.f	n.f	n.f	n.f	n.f	n.f	n.f
A14	Ethyl nonanoate	200	Fruity, coconut [21]	0.14 ± 0.00 ^eA^	0.45 ± 0.05 ^d^	0.56 ± 0.01 ^bc^	0.54 ± 0.03 ^c^	0.64 ± 0.09 ^b^	0.83 ± 0.01 ^a^	0.25 ± 0.01 ^A^	0.29 ± 0.01 ^A^	0.33 ± 0.03 ^A^	0.37 ± 0.00 ^A^	0.37 ± 0.02 ^A^
F2	2-Methoxy-4-vinylphenol	0.012	Clove, curry [26]	65,499.99 ± 1397.30 ^aA^	79,844.20 ± 22,279.51 ^a^	84,661.21 ± 19,881.68 ^a^	87,206.22 ± 1653.96 ^a^	94,087.90 ± 5059.04 ^a^	89,996.50 ± 1964.07 ^a^	64,961.68 ± 579.59 ^A^	59,333.86 ± 2778.46 ^A^	59,387.56 ± 8320.75 ^A^	58,178.18 ± 7381.30 ^A^	56,011.51 ± 1518.48 ^A^
A23	Ethyl hexadecanoate	1500	Wax [21]	0.03 ± 0.00 ^aA^	0.12 ± 0.09 ^a^	0.14 ± 0.03 ^a^	0.19 ± 0.03 ^a^	0.28 ± 0.03 ^a^	0.27 ± 0.12 ^a^	0.05 ± 0.00 ^A^	0.04 ± 0.01 ^A^	0.06 ± 0.00 ^A^	0.10 ± 0.03 ^A^	0.05 ± 0.02 ^A^
A22	Ethyl tetradecanoate	2000	Sweet, creamy [21]	0.01 ± 0.00 ^aA^	0.02 ± 0.01 ^a^	0.03 ± 0.00 ^a^	0.03 ± 0.01 ^a^	0.05 ± 0.00 ^a^	0.05 ± 0.02 ^a^	0.01 ± 0.00 ^A^	0.01 ± 0.00 ^A^	0.01 ± 0.00 ^A^	0.02 ± 0.01 ^A^	0.01 ± 0.00 ^A^
A6	Hexyl acetate	2	Pear [28]	153.73 ± 2.43 ^aB^	64.89 ± 6.86 ^b^	51.75 ± 2.07 ^c^	27.01 ± 0.12 ^d^	18.58 ± 2.17 ^e^	22.94 ± 0.45 ^de^	195.62 ± 1.12 ^A^	171.75 ± 6.14 ^AB^	178.11 ± 15.19 ^AB^	158.38 ± 17.99 ^B^	158.82 ± 20.94 ^B^
E4	4-Methyl-2-phenyl-2-pentenal	n.f	n.f	n.f	n.f	n.f	n.f	n.f	n.f	n.f	n.f	n.f	n.f	n.f
A21	Ethyl dodecanoate	1500	Rose, honey [21]	0.23 ± 0.05 ^aC^	0.11 ± 0.03 ^a^	0.11 ± 0.01 ^a^	0.13 ± 0.02 ^a^	0.15 ± 0.00 ^a^	0.15 ± 0.04 ^a^	0.46 ± 0.01 ^B^	0.53 ± 0.04 ^AB^	0.61 ± 0.04 ^A^	0.62 ± 0.04 ^A^	0.45 ± 0.12 ^B^
C5	(*E*)-β-Farnesene	87	Floral [22]	1.13 ± 0.02 ^aB^	0.63 ± 0.17 ^a^	0.55 ± 0.03 ^a^	0.45 ± 0.02 ^a^	0.42 ± 0.00 ^a^	0.31 ± 0.01 ^a^	1.54 ± 0.03 ^A^	1.52 ± 0.05 ^A^	1.43 ± 0.23 ^A^	1.67 ± 0.01 ^A^	1.14 ± 0.20 ^B^
C6	*α*-Santalol	n.f	n.f	n.f	n.f	n.f	n.f	n.f	n.f	n.f	n.f	n.f	n.f	n.f
A5	Ethyl hexanoate	14	Apple, peach [21]	333.13 ± 8.99 ^aA^	213.18 ± 17.07 ^d^	249.20 ± 8.73 ^c^	264.83 ± 10.33 ^c^	288.51 ± 3.84 ^b^	303.90 ± 2.49 ^b^	424.14 ± 1.99 ^A^	419.35 ± 5.72 ^A^	454.83 ± 47.58 ^A^	505.97 ± 36.97 ^A^	561.69 ± 29.27 ^A^
B5	1-Heptanol	200	Fruity, musty [21]	0.81 ± 0.01 ^abC^	0.75 ± 0.07 ^b^	0.82 ± 0.01 ^ab^	0.81 ± 0.01 ^ab^	0.76 ± 0.01 ^b^	0.87 ± 0.07 ^a^	1.01 ± 0.02 ^B^	1.02 ± 0.04 ^B^	1.04 ± 0.01 ^B^	1.11 ± 0.02 ^A^	1.12 ± 0.03 ^A^
B9	Phenylethyl Alcohol	1100	Rose [24]	3.83 ± 0.04 ^aA^	2.99 ± 0.18 ^bc^	2.86 ± 0.34 ^bc^	3.09 ± 0.24 ^b^	2.99 ± 0.17 ^bc^	2.69 ± 0.09 ^c^	4.26 ± 0.14 ^A^	3.98 ± 0.03 ^A^	3.73 ± 0.50 ^A^	3.78 ± 0.10 ^A^	3.93 ± 0.05 ^A^
E1	1,1-Diethoxyethane	50	Green, fruity [19]	7.48 ± 1.12 ^aBC^	3.03 ± 0.64 ^c^	4.78 ± 0.59 ^b^	2.62 ± 0.03 ^c^	3.10 ± 0.02 ^c^	3.65 ± 0.27 ^c^	10.42 ± 1.79 ^A^	9.29 ± 0.28 ^AB^	6.20 ± 2.95 ^C^	11.58 ± 0.79 ^A^	11.88 ± 0.72 ^A^
C12	1-(1,4-Dimethylcyclohex-3-en-1-yl)ethan-1-one	n.f	n.f	n.f	n.f	n.f	n.f	n.f	n.f	n.f	n.f	n.f	n.f	n.f
C8	*α*-Farnesene	87	Floral [22]	1.20 ± 0.03 ^aA^	2.45 ± 0.64 ^a^	2.10 ± 0.30 ^a^	1.27 ± 0.02 ^a^	0.89 ± 0.05 ^a^	0.70 ± 0.04 ^a^	2.70 ± 0.03 ^A^	4.02 ± 0.22 ^A^	4.08 ± 0.30 ^A^	4.55 ± 0.17 ^A^	3.08 ± 0.83 ^A^
A8	Ethyl 2-hexenoate	n.f	n.f	n.f	n.f	n.f	n.f	n.f	n.f	n.f	n.f	n.f	n.f	n.f
C11	Eudesma-1,4(15),11-triene	n.f	n.f	n.f	n.f	n.f	n.f	n.f	n.f	n.f	n.f	n.f	n.f	n.f
C7	(Z)-epi-β-Santalol	n.f	n.f	n.f	n.f	n.f	n.f	n.f	n.f	n.f	n.f	n.f	n.f	n.f
A13	2-Phenylethyl acetate	250	Honey, sweet [24]	2.08 ± 0.02 ^aA^	2.10 ± 0.20 ^a^	2.00 ± 0.16 ^a^	1.34 ± 0.26 ^a^	1.01 ± 0.15 ^a^	0.77 ± 0.05 ^a^	2.47 ± 0.11 ^A^	2.22 ± 0.00 ^A^	2.07 ± 0.22 ^A^	2.17 ± 0.06 ^A^	2.21 ± 0.06 ^A^

Note: Odor thresholds are expressed in μg/L. Estimated odor activity values (EOAVs) are dimensionless. n.f: No threshold was found; n.d: Not detected. CK, control group without enzyme treatment; C1–C5, cellulase-treated groups; G1–G5, β-glucosidase-treated groups. Lowercase letters indicate a significant difference between the cellulase-treated group and the control group. Uppercase letters indicate a significant difference between the β-glucosidase-treated group and the control group.

**Table 4:** Entropy-derived weights of evaluation indicators for the cellulase-treated group.

Indicator	Information Entropy (e)	Information Utility (d)	Weight (w)
b*	0.8752	0.1248	13.6143%
Fruit aroma	0.8416	0.1584	17.2790%
Floral aroma	0.9044	0.0956	10.4290%
Overall balance	0.8420	0.1580	17.2320%
Total flavonoids	0.9001	0.0999	10.8918%
FRAP	0.8856	0.1144	12.4748%
Transmittance	0.8342	0.1658	18.0790%

Note: b* represents the CIELAB color coordinate on the blue–yellow axis.

**Table 5:** Entropy-derived weights of evaluation indicators for the β-glucosidase-treated group.

Indicator	Information Entropy (e)	Information Utility (d)	Weight (w)
b*	0.8720	0.1280	11.0655%
Fruit aroma	0.8861	0.1139	9.8494%
Floral aroma	0.8914	0.1086	9.3931%
Overall balance	0.8576	0.1424	12.3131%
Total flavonoids	0.5991	0.4009	34.6687%
FRAP	0.8357	0.1643	14.2043%
Transmittance	0.9016	0.0984	8.5059%

Note: b* represents the CIELAB color coordinate on the blue–yellow axis.

**Table 6:** TOPSIS evaluation results for the cellulase-treated group.

	Distance to the Positive Ideal Solution (D+)	Distance to the Negative Ideal Solution (D−)	Relative Closeness Coefficient (C)	Rank
CK	0.266	0.245	0.480	4
C1	0.080	0.321	0.800	1
C2	0.158	0.290	0.647	2
C3	0.213	0.229	0.518	3
C4	0.234	0.209	0.472	5
C5	0.289	0.240	0.454	6

Note: CK, control group without enzyme treatment; C1–C5, cellulase-treated groups.

**Table 7:** TOPSIS evaluation results for the β-glucosidase-treated group.

	Distance to the Positive Ideal Solution (D+)	Distance to the Negative Ideal Solution (D−)	Relative Closeness Coefficient (C)	Rank
CK	0.411	0.120	0.226	6
G1	0.269	0.247	0.479	3
G2	0.355	0.202	0.363	5
G3	0.362	0.212	0.369	4
G4	0.207	0.266	0.562	2
G5	0.199	0.367	0.648	1

Note: CK, control group without enzyme treatment; G1–G5, β-glucosidase-treated groups.

## Data Availability

The original contributions presented in this study are included in the article. Further inquiries can be directed to the corresponding authors.
